# Minimally Invasive Surgery, Implantable Sensors, and Personalized Therapies

**DOI:** 10.18502/jovr.v15i4.7792

**Published:** 2020-10-25

**Authors:** Kevin Gillmann, Kaweh Mansouri

**Affiliations:** ^1^Glaucoma Research Center, Montchoisi Clinic, Swiss Visio, Lausanne, Switzerland; ^2^Department of Ophthalmology, University of Colorado School of Medicine, Denver, CO, USA

**Keywords:** Glaucoma, MIGS, Quality of Life, Telemetry, Eyemate, Bimatoprost SR

## Abstract

Glaucoma management has changed dramatically over the last decades, through clinical advances and technological revolutions. This review discusses the latest innovations and challenges faced in the field around three major axes: minimally-invasive glaucoma surgery (MIGS), implantable sensors and injectable therapeutics.

Indeed, the vast number of recently developed MIGS techniques has not only provided clinicians with a wide range of therapeutic options, but they have also enabled them to adjust their therapies more finely which may have contributed a more patient-centric decision-making process. Yet, despite considerable advances in the field, the wide heterogeneity in clinical trial designs blurs the surgical outcomes, specificities and indications. Thus, more high-quality data are required to make the choice of a specific MIGS procedure more than an educated guess. Beyond the scope of MIGS, the potential of IOP telemetry for self-assessment of IOP-control through implantable sensors is developing into a real option for clinicians and an empowering opportunity for patients. Indeed, providing patients with direct feedback enables them to take control and have a clearer representation of their care, in turn leading to a better control of the disease. However, there are potential issues with self-monitoring of IOP, such as increased anxiety levels induced by measured IOP fluctuations and peaks, leading to patients self-treating during IOP spikes and additional office visits. Furthermore, the advent of implantable therapeutics may soon provide yet another step towards personalized glaucoma treatment, by offering not only an efficient alternative to current treatments, but also a therapeutic option that may better adapt to patients’ lifestyle.

After several decades of relative stagnation through the last century, glaucoma has now entered what many view as a golden age for the specialty. Like every revolution, this one brings its fair share of uncertainty, clinical questioning and uneasy periods of adaptation to ever-changing expectations. Yet, while it is impossible to guess what the landscape of glaucoma surgery will be like in ten or fifteen years, data suggest a bright outlook both for patients and clinicians.

##  INTRODUCTION

Glaucoma is a leading cause of irreversible blindness, and it is estimated that by 2040, over 110 million people will suffer from glaucoma globally.^[1]^ To face the increasing burden of glaucoma, its management has changed dramatically over the last decades, through clinical advances and technological revolutions. Indeed, while the 1990s were the decade of glaucoma drainage devices (GDD) and novel topical therapeutic agents, the new millennium has witnessed an unprecedented growth in treatment options through the introduction and integration of minimally invasive glaucoma surgeries (MIGS), and the surgical applications of cross-disciplinary innovations. This review discusses the most remarkable innovations of the last decade and explores how they may affect the future of glaucoma surgery.

##  METHODS

Multiple literature searches were conducted in preparation of this review. Searches were conducted on PubMed and Google Scholar, using the following keywords. For section A on MIGS technologies: glaucoma surgery, MIGS, trabecular bypass, suprachoroidal drainage, subconjunctival filtration, Trabectome, Kahook Dual Blade, Trabeculotomy, Canaloplasty, Hydrus Microstent, iStent, CyPass, Cyclophotocoagulation, Preserflo, XEN Gel Stent, glaucoma physiology, aqueous outflow. For section B on intraocular sensors: IOP variations, 24-hour IOP, dynamic tonometry, telemetry, intraocular sensor, Eyemate. For section C on implantable and injectable therapeutics: glaucoma pharmacology, prostaglandin, glaucoma treatment compliance, glaucoma drug delivery, intracameral implant, bimatoprost SR, Durysta. Furthermore, references from the reviewed articles and textbooks were considered for inclusion within this review, regardless of their publication date or language.

##  RESULTS

### a. Minimally Invasive Glaucoma Surgery

The first-line treatment for open-angle glaucoma has long been topical pharmaceutical therapies or laser treatments. Yet, until relatively recently, the only alternative when these failed was filtering surgery. With popularization of anti-metabolites in glaucoma surgery, from the start of the 1990s, filtering surgery evolved into highly effective procedures, with a reported intraocular pressure (IOP) reduction as high as 50%.^[2]^ However, this evolution was associated with an increase in severe adverse events such as chronic hypotony, bleb leak, or endophthalmitis, with a rate of late complications in excess of 30% in some reports.^[3]^ MIGS were designed to bridge the gap between medical or laser therapies and more invasive filtering surgeries in mild to moderate glaucoma. By essence, MIGS are meant to have an extremely favorable safety profile ensuring prompt postoperative recovery; however, the amount of IOP reduction is not as high as traditional filtering surgery.^[4]^ Through the array of available techniques, MIGS have not only provided clinicians with a wider range of therapeutic options, but they have also enabled them to adjust their therapies more finely which may have contributed to a more patient-centric decision-making process. However, it can be a bit overwhelming to choose from such a large armamentarium, especially in the absence of evidence-based criteria.

### Approaches

**Figure 1:**
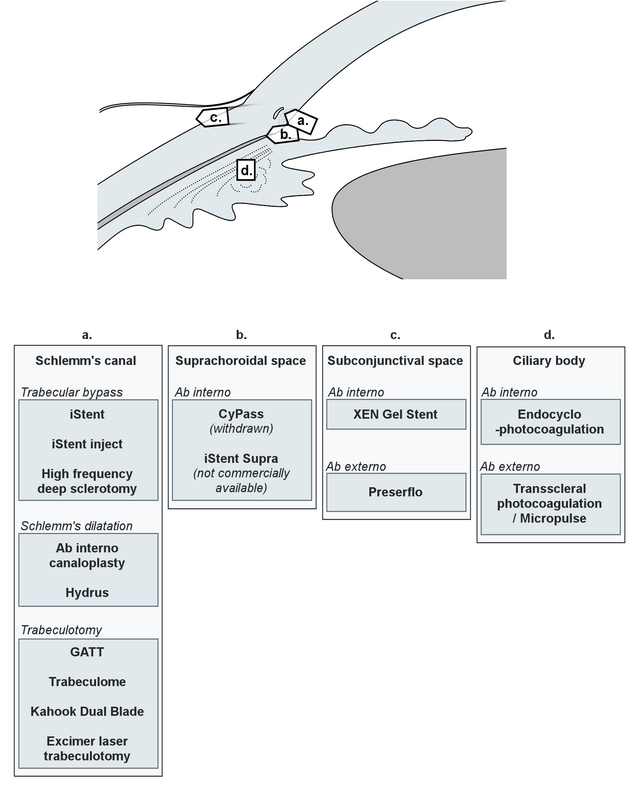
Illustration of the different classes of minimally invasive glaucoma surgery, their anatomical relationship with the ocular structures (top), and examples of implants and techniques available in each category.

MIGS are generally classified based on their anatomical sites of action. Their mechanisms can focus on: (1) Schlemm's canal, (2) Suprachoroidal space, (3) Subconjunctival space, and (4) Ciliary body. Each class of MIGS has its own advantages and limitations, and several different devices or techniques coexist within most categories. Most of these options differ in their dimensions, but in some instances, there may be some more fundamental technical variations. The main classes of MIGS are summarized in Figure 1.

**Figure 2:**
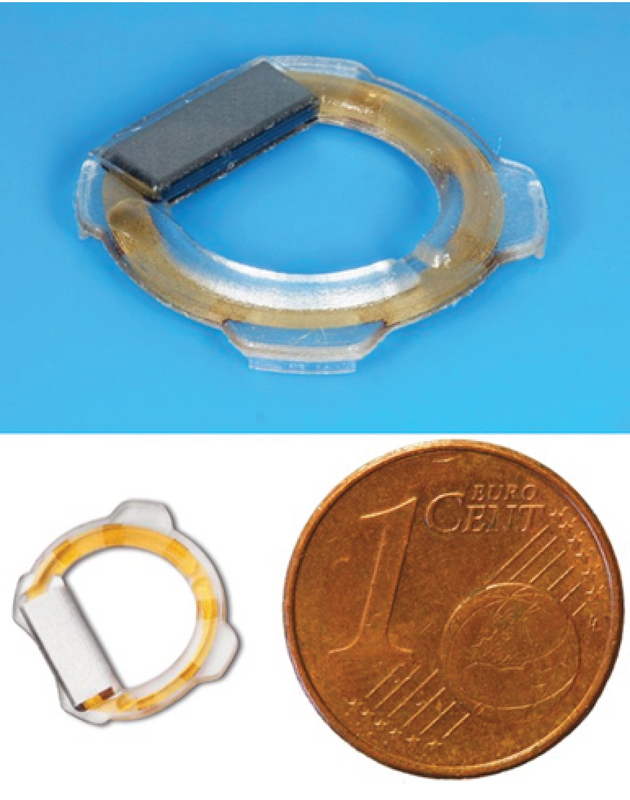
Eyemate intraocular sensor (top) and a size comparison with a eurocent coin.

**Figure 3:**
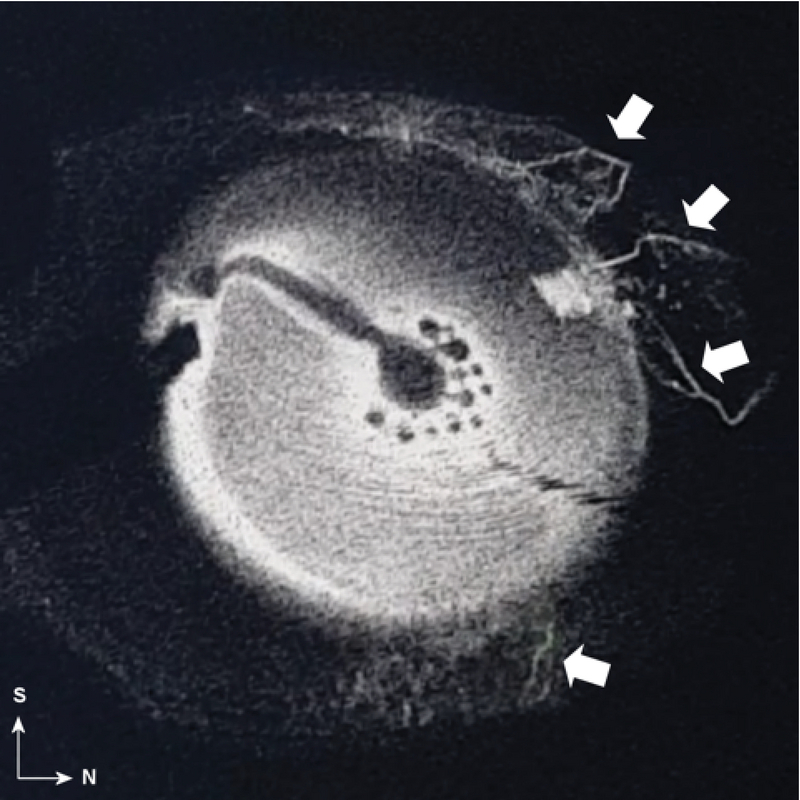
Intraoperative aqueous angiography showing the collector channels (arrows). S, Superior; N, Nasal

**Figure 4:**
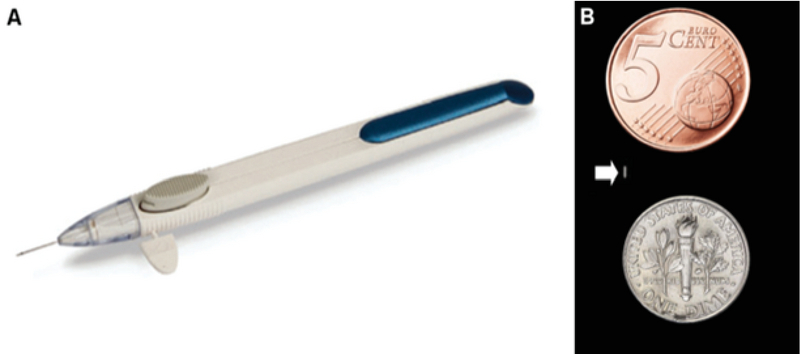
Bimatoprost SR injector (A) and the bimatoprost implant, compared with the size of a dime and a eurocent coin (B).

#### Schlemm's canal: Trabecular meshwork bypass and Schlemm's canal dilatation

The trabecular or conventional pathway is the principal route for aqueous humor outflow in a physiological condition. Aqueous percolates through the trabecular meshwork into Schlemm's canal, before entering a wide network of vessels through the collector channels. In primary open-angle glaucoma, however, trabecular meshwork outflow resistance increases, possibly in response to extracellular matrix changes, the etiology of which is still mostly unknown.^[5,6]^ Furthermore, in the early 1960s, Grant showed how ab interno 360° removal of the trabecular meshwork resulted in a 75% reduction of the total resistance in enucleated eyes at an IOP of 25 mmHg.^[7,8]^ Bypassing a site of increased outflow resistance (often considered the primary site of resistance) and enhancing the main physiological outflow pathway are two of the principles underlying the rationale of trabecular meshwork bypass or ablation. This class of MIGS aims to reduce outflow resistance and IOP by facilitating aqueous drainage into Schlemm's canal either by bypassing the trabecular meshwork via some stent devices or by merely removing all or a portion of the trabecular meshwork. Several variations of stent devices and trabecular meshwork ablation techniques exist.

However, recent studies have suggested that contrary to the common belief that the main site of glaucoma resistance lies within the juxtacanalicular trabeculum, the IOP elevation observed in primary open-angle glaucoma is more accurately caused by a combination of three equally determinant factors: (1) loss of permeability of the entire thickness of the trabecular meshwork, (2) collapse of Schlemm's canal, and (3) downstream resistance, notably with the closing of collector channel entrances.^[11,12,13]^ This was further supported by the finding that Schlemm's canal dilatation was positively correlated with the magnitude of IOP reduction.^[9]^ It was, therefore, hypothesized that Schlemm's canal increased volume is associated with the stretching of its walls, which in turn causes the opening of pressure-dependent collector channels, leading to aqueous outflow.^[10]^ Based on these observations, another subcategory of MIGS specifically targets Schlemm's canal, with the aim of restoring a healthy Schlemm's canal function and opening closed collecting channels. Two approaches were used to dilate Schlemm's canal: the mechanical dilatation using a temporary or resorbable medium and the use of a permanent implantable scaffold.

Despite theoretically different approaches, these two subcategories of MIGS are, in effect, physiologically related. Indeed, while the latter group directly targets Schlemm's canal to cause its dilatation with aqueous humor and restore distal outflow capacity, studies have shown that the former group, while merely bypassing the trabecular meshwork, produces a similar effect. Indeed, it has been reported that the magnitude of IOP reduction following trabecular meshwork bypass implantation was directly correlated to the dilatation of Schlemm's canal.^[11]^ Furthermore, aqueous angiography techniques have shown that, beyond their effects on Schlemm's canal, trabecular bypass devices could increase collector channel outflow.^[12]^ The limitation of both approaches is that they do not address any resistance that may be distal to the collector channels' openings. Therefore, there is a floor effect to IOP reduction depending on distal outflow resistance.

#### Aqueous angiography: The road to personalized surgery

The role of distal outflow resistance in IOP reduction indicates the importance of targeting collector channels with MIGS. It was reported that trabeculotomies performed in the nasal hemisphere, where the concentration of collector channels is denser, increases the outflow more than trabeculotomies performed in the temporal hemisphere,^[13]^ but more recent research suggests a more nuanced reality. Huang et al have designed a new imaging technique allowing collector channel visualization using intraoperative aqueous angiography. Their first report suggested that targeting an area deprived of collector channel outflow could recruit new, previously closed, channels.^[14]^ Figure 2 shows collector channels identified with aqueous angiography. It could therefore be speculated that locating collector channels prior to MIGS or filtering surgery may result in a more targeted treatment. In the absence of dedicated comparative studies, however, the question remains controversial and most MIGS procedure continue to be performed supero-nasally, both for practical reasons and to target more collector channels.

#### Suprachoroidal space: Suprachoroidal shunts

The physiological proportion of aqueous humor draining through the suprachoroidal space is subject to debate due to the lack of techniques available to measure uveoscleral flow, but estimates range between 4% and 60%.^[15,16]^ It is, however, accepted that aging is responsible for a marked reduction in uveoscleral outflow.^[17]^ This outflow pathway is created by a combination of relative ciliary body permeability, which is believed to be the site of main resistance in the uveoscleral pathway,^[18]^ and the existence of a hydrostatic pressure gradient through the anterior chamber, the supraciliary space, and the suprachoroidal space.^[19]^ Such a negative gradient is believed to be produced by the rapid absorption of aqueous from the suprachoroidal space into the large and dense choroidal vasculature.^[20,21]^ Another characteristic of the uveoscleral pathway is that it is relatively pressure-independent and was shown to have a constant effect between 4 and 35 mmHg.^[22]^

These last two characteristics suggest that exploiting uveoscleral pathway may theoretically make up for some of the conventional pathway limitations: the risk of distal resistance and the floor effect. However, devices targeting this pathway can be expected to have a whole different risk profile to trabecular bypass devices. Indeed, the potentially greater outflow capacity of this approach could, in theory, be associated with higher risks of hypotony and choroidal detachment, especially in patients with a long history of prostaglandin therapy. While the cases are too rare to warrant for a prospective study, there have been anecdotal cases suggesting that patients who were chronically treated with prostaglandins may be at a higher risk of developing choroidal pathologies.^[28,29,30,31]^ This may be related to the effect of prostaglandins, reducing collagens within the uveoscleral pathway.^[23]^ Furthermore, from a practical point of view, the suprachoroidal space may be less readily accessible and visualizable by a surgeon than the trabeculum.

While there is no commercially available MIGS relying on suprachoroidal drainage, some new devices are under development and sound clinical data is available on a previously commercialized device. Therefore, we will discuss the case of this device, some characteristics of which may be comparable to future devices of the same category.

#### Subconjunctival space: Subconjunctival filtration

Contrary to the trabecular and the uveoscleral approaches, subconjunctival filtration does not seek to enhance or increase a physiological pathway. Instead, it relies on the creation of an artificial canal between the anterior chamber and the subconjunctival space, typically through a stent. This process results in an iatrogenic filtration bleb from which aqueous humor diffuses into the surrounding subconjunctival tissue and is eventually reabsorbed into subconjunctival capillaries.^[24]^

The idea of subconjunctival filtration is not new and stems from the anterior sclerectomy technique designed by De Wecker in 1858.^[25]^ While modern-day trabeculectomies and deep sclerectomies have considerably refined the technique, the use of the subconjunctival pathway has survived. Like trabeculectomy, the success of subconjunctival MIGS procedure depends on the persistence of a healthy filtering bleb. Therefore, these MIGS share many similarities with filtering surgeries, in terms of risks and advantages. One of the main advantages of subconjunctival filtration is precisely that it does not impact any of the physiological outflow pathways, and as such, preserves any remaining physiological filtration. Another significant advantage of these techniques is that they do not rely on episcleral venous pressure or suprachoroidal pressure gradients to achieve filtration. Instead, their filtration capacity is only dependent on the outflow resistances of the stent and the subconjunctival space. Therefore, they can potentially achieve lower IOPs than physiological approaches.

The outflow resistance of the subconjunctival space, however, is very much patient dependent and can be difficult to predict. A significant factor recognized to influence resistance is conjunctival scarring and fibrosis, which results in the failure of filtering surgery.^[26]^ The pathophysiology of fibrosis is complex, but growth factors and cytokines expressed in inflammatory cells are clear culprits.^[27]^ This is particularly problematic in glaucoma patients when inflammation is exacerbated through four mechanisms: (1) the predisposition of patients to conjunctival fibrosis through long-term use of topical prostaglandins or toxic preservative, both of which were associated with local inflammation,^[28,29]^ (2) the surgical procedure itself, (3) subconjunctival flow, by itself, constitutes a persistent mechanical stress to local tissue, which was shown to translate into pro-inflammatory biochemical signals,^[39,40,41]^ and (4) the mere presence of aqueous humor in the subconjunctival space, where it is not naturally present, was shown to promote tissue fibrosis. Some components, particularly TGF-b and VEGF-A, present at increased levels in the aqueous humor of glaucoma patients are believed to be responsible for subconjunctival fibrosis.^[30,31]^ While both VEGF antagonists and Rho-kinase inhibitors were suspected to be beneficial in the context of bleb surgery, they have so far failed to demonstrate clear superiority or to translate into clinical practice,^[32,33]^ and, to date, the clinical recommendations with regards to inflammation mediation are the preoperative washout from pro-inflammatory topical medications and the prolonged postoperative use of topical steroids. This point, however, remains the major impediment to sustainable subconjunctival filtration. With this regard, MIGS may have a role to play in reducing the amount of inflammation caused by subconjunctival procedures.

Further risks common to all bleb-creating procedures include bleb dysesthesia, bleb leaks, blebitis, and bleb-related endophthalmitis. These complications can be common and some authors have reported rates of bleb interventions in excess of 50% following XEN implantations.^[34]^ Hypotony is another inherent risk of bypassing physiologic outflow pathway, but this risk can theoretically be mediated by the adjustment of devices' internal dimensions to create specific levels of outflow resistance.^[35]^ Finally, contrary to traditional filtration surgery, prospective studies and occasional case reports have highlighted a risk of stent displacement and occlusion, which are inherent to the placement of an artificial stent.^[36,37]^

#### Ciliary body: Reduction of aqueous humor production

The ciliary body is site of aqueous production. Reducing aqueous humor production is a logical alternative to enhancing aqueous outflow to lower IOP. Cyclophotocoagulation consists of using a laser to selectively deliver thermal energy to the pigmented tissues of the ciliary body and induce tissue coagulative necrosis.^[38]^ Historically, the technique that emerged in the 1930s as cyclodiathermy has long been exclusively indicated for refractory glaucoma and painful blind eyes. This was mostly due to the relatively high risk of intense and chronic postoperative inflammation, pain, hypotony, vision loss, and phthisis.^[39,40]^ However, recent innovations have allowed for more targeted treatments and less collateral tissue necrosis, leading to reduced complication rates and better safety profiles. This has led to cyclophotocoagulation's gradual acceptance for the treatment of milder forms of glaucoma, and to some surgeons considering it a MIGS. The main theory underlying this change in practice is that the rates and severity of complications following cyclophotocoagulation are directly related to the total amount of energy used during the procedure.^[41]^ However, despite a clear reduction in the rates of complications over the last decades, the risk of permanent visual loss to a sighted eye remains non-negligible,^[42,43]^ and a recent Cochrane review concluded that there was still insufficient evidence to conclude positively on the effectiveness and safety of cyclophotocoagulation in non-refractory glaucoma.^[44]^

Furthermore, it has been speculated that the significant perilimbal conjunctival inflammation and scarring produced by transscleral cyclophotocoagulation could affect the outcome of subsequent filtering surgeries, casting further doubt over the indications of this type of treatment as an initial procedure.

#### b. Intraocular sensors

With such an array of techniques designed specifically to lower IOP, pressure control has become the key element of surgical outcomes and the cornerstone of glaucoma care as a whole. Innovation, however, has been slower when it comes to IOP measurement techniques, and the gold standard technique has essentially remained the same since 1950, when Goldmann applanation tonometery (GAT) was introduced.^[45]^ GAT relies on the Imbert-Fick principle to estimate IOP based on the force that is required to flatten the 3.06 mm diameter area corresponding to the tip of the Goldmann prism. Yet, this technique is widely regarded as imperfect and studies have pointed out its flaws both in terms of design and concept. Not only is GAT relatively imprecise, its instant nature also fails to reflect the complexity of real-life IOP variations.^[58,59,60]^ Indeed, over the last decades, there has been ample evidence that individuals' IOP are far from being static and fluctuate widely over the course of 24 hours and through the year, and even suggestions that IOP variations may play a significant role in glaucoma progression regardless of their absolute values.^[61,62,63,64,65,66]^

#### Eyemate

While several companies are actively working on IOP-monitoring sensor prototypes, the German company Implandata introduced the first implantable continuous monitoring device. It was CE-certified in 2017 for use in primary open-angle glaucoma patients, and is composed of three devices: the implantable sensor (or wireless intraocular transducer [WIT]), the handheld reader unit, and the wireless module.^[67,68,69]^ The sensor itself is implanted in the ciliary sulcus during routine cataract surgery and is designed to stay in the patient's eye indefinitely. To offer options to patients regardless of their lens status or anterior chamber pathologies, another suprachoroidal approach was developed and is currently being assessed. Figure 3 shows a picture of the Eyemate sensor.

Technically, the WIT consists of eight miniature pressure sensor cells, a temperature sensor, an identification encoder, an analog-to-digital encoder, and a telemetry unit into a single microelectromechanical system (MEMS). The MEMS is attached to a gold circular antenna and the entire device is encapsulated in implantation-grade silicone. It weighs 0.1 g and comes in three sizes with a varying external diameter of 11.3 mm, 11.7 mm, or 12.1 mm, in order to accommodate varying sulcus diameter. It has an internal diameter of 7 mm and an overall thickness of 0.5 mm, while the thickness at the sensor area is 0.9 mm. Each MEMS pressure sensor cell is made of two miniature parallel plates: a thin flexible plate that indents with changes in IOP and a thicker rigid base plate integrated with an A/D converter for the digitalized pressure information. With changes in IOP, the thin plate is mechanically defected. As the distance between the two plates varies, a corresponding analogue signal is generated and then converted to a digital signal that is transmitted externally by radiofrequency.^[46]^

The handheld reader unit, that resembles a television remote control, receives the digital data and visually displays the IOP values on its LED display. The reader and the intraocular transponder unit must be within 5 cm of each other before a button is pressed on the reader to activate the electromagnetic coupling sequence and the two units can correspond with each other. This is all the cooperation required from the patient. The sensor does not require a battery. The handheld reader is battery powered and supplies the WIT externally through electromagnetic inductive coupling at the time of communication. The sensor can conduct up to 10 IOP measurements per second and there are a range of settings that allow for automatic monitoring at variable intervals. The base unit can store up to 3000 IOP measurements, and additional memory modules can be added to the reader device. The wireless module can be installed to automatically download all measured data to a cloud-based server, allowing the clinical provider easy and instantaneous access to the data. The ophthalmologist receives the patient's IOP measurements and can easily create a tension profile, detect dips and peaks during diurnal and nocturnal IOP fluctuations, and recognize situations that require adjustments of the therapy.

In 2011, the ARGOS-01 study investigated the safety of the Eyemate and the accuracy of its IOP measurements. Six patients with POAG underwent cataract surgery and ciliary sulcus placement of the sensor. The IOP sensor was well tolerated by all the patients, despite four of them developing self-limited sterile anterior chamber inflammation. No difference in endothelial cell count and central corneal thickness was observed after 12 months in any of the patients. All patients were able to perform self-tonometry as instructed, and telemetric IOP values correlated well with GAT measurement, except for one patient who recorded negative values throughout the study.^[47]^

Since the original study, other reports illustrated how close self-monitoring could detect non-symptomatic IOP variations that would otherwise go unnoticed. Abnormal self-measurements can prompt the patients to obtain more IOP measurements and search for repetitive patterns. In a published case report by Rüfer et al, a patient even found a rare connection between the administration of his dorzolamide eyedrops and the subsequent IOP rises before he received any medical input.^[48]^ This suggests that placing the patient in charge and providing them with the means to easily and accurately self-monitor could improve glaucoma care by detecting IOP aberrations earlier and eliciting causative factors with more ease. The increased frequency of IOP measurement associated with the democratization of such sensors is likely to lead to an increased incidence of abnormal pressure readings. While this may represent crucial information on a patient's individual IOP control, more research will be needed to determine the clinical relevance of every fluctuation captured through continuous monitoring.

#### c. Implantable and injectable therapeutics

Despite all these innovations, the most common first-line treatment for open angle glaucoma remains pharmacological, and often consists of a topical prostaglandin analog (PGA). Numerous studies have confirmed their potential to achieve substantial IOP reductions for up to 48 hours, providing both diurnal and nocturnal effect, contrary to topical beta-blockers that were proven to be somewhat inefficient at night. This class of medications act by primarily increasing uveoscleral outflow and, to some extent, enhancing trabecular outflow. While PGA is a generally well-tolerated medication, a number of side effects are commonly reported, including cystoid macular edema, excessive eyelash growth, iris pigmentation, allergic reactions, and conjunctival hyperhemia and scarring. The latter of which was associated with increased risk of surgical failure in subsequent filtering procedures. Beyond these adverse effects, topical PGA suffer the same problems as all self-administered topical medications.^[49]^

There is ample data, both self-reported and from pharmacies, indicating that adherence with topical anti-glaucoma treatments is generally poor. Indeed, American studies have estimated that as many as 27% of all prescribed medications are simply not purchased by the patients, and nearly half of all glaucoma patients miss over 25% of their treatment doses.^[74,75,76]^ Unsurprisingly, poor compliance is associated with worse IOP control and disease progression. In addition, even when topical medications are administered, patients' instillation techniques can be poor and lead to a further decrease in treatment efficacy. To remedy these universal issues inherent to self-administered eye drops, several drug delivery approaches are being developed and will soon become available to the glaucoma specialists. They include options as varied as drug-eluting contact lenses or periocular rings, topical nebulizers releasing microdroplets of the active ingredient directly on the surface of the cornea, or subconjunctival injections. One of them, an intracameral bimatoprost-releasing implant, has already reached stage 3 clinical trials and is showing promising results.^[50]^

It consists of a biodegradable matrix designed to be injected into the anterior chamber, where it gradually releases 15 μg of sustained-release bimatoprost over a period of up to six months. Figure 4 shows the implant. In recent studies, the efficacy of the implant was comparable to that of daily instillation of the topical medication, with no observable side effects.^[51]^ This mode of administration presents several clear advantages. Firstly, with patients only having to attend a bi-annual appointment to receive their treatments, it would considerably reduce the potential for missed doses compared to topical therapies. Secondly, the seemingly reduced contact and effect of bimatoprost on ocular surface may decrease the prevalence of long-term side effects and the negative impact of prolonged treatments on subsequent filtering surgeries. Thirdly, and interestingly, contrary to topical instillation, no ceiling effect was identified for intracamerular instillation of bimatoprost. It has been speculated that it may be due to a direct lowering effect of intraocular bimatoprost on episcleral venous pressure. Finally, after several intracameral injections, the IOP-lowering effect of the treatment appeared to persist considerably longer than the six-month lifespan of the implant, leading to prolonged periods of treatment free reduced IOP.^[52]^ The advent of implantable or injectable therapeutics may soon provide yet another step in the glaucoma treatment ladder.

#### Opinion

The traditional landscape of glaucoma management has changed dramatically over the last decade, with the development of a large array of novel surgical techniques. While a surge in attention, investment and innovation and, eventually, treatment options foretells a bright future for the sub-specialty, at a clinical level, it raises the questions of patient-centered treatment choices and evidence-based decisions. Indeed, one of the advantages of such a heterogenous range of surgical options is the chance to tailor therapy in an individualized manner. High quality data are required to make this choice more than an educated guess. To provide some objective criteria in the assessment of glaucoma surgery, and to guide innovation, the “10-10-10 Goal” was set. According to these criteria, the ideal surgical technique would take less than 10 min to perform, be able to consistently achieve IOPs below 10 mmHg, and last >10 years without any significant complications.

With procedures typically taking between 15 and 30 min to perform, MIGS have managed to significantly reduce surgical times. While this represents a 50% reduction from most traditional filtering procedure or glaucoma drainage device implantations, MIGS are yet to provide us with a simple enough procedure to be consistently carried out in <10 min by the average glaucoma surgeon. In terms of IOP reduction potential, considering that the average candidate for MIGS surgery has a preoperative IOP in the 20–25 mmHg range, it would require a 50–60% reduction to achieve postoperative pressures under the 10-mmHg threshold. In the literature, only rarely did some MIGS provide IOP reduction of 50% or more. Furthermore, the few incidences of IOP reductions >50% could not be replicated, and the same surgical techniques showed more modest effects in alternative studies. It does, however, appear that the sub-conjunctival approach is more likely than other categories of MIGS to achieve IOPs in the low-teens. But in this context of intense innovation, new technologies will likely appear and reshuffle the cards in the years to come, including new devices offering variations on existing techniques, or all-new approaches such as drug-coated devices or ocular surface shunts. Finally, it is, at present, difficult to assess the sustainability of MIGS efficiencies. Since most MIGS have been commercially available for <5 years, there is a general lack of long-term data in the field, but knowledge is slowly accruing. This aim of longevity, however, should prompt us to design sound clinical trials early on, in order to obtain not only extensive, but also reliable long-term data.

Overall, reviews confirm the efficiency of most MIGS compared to standalone phacoemulsification, and the reported rates of complications also compare favorably with traditional filtering surgeries. But to be clinically advisable, a procedure needs not only be safe, but also to prove its non-inferiority to commonly accepted alternatives. However, there are only few studies comparing different MIGS techniques, especially considering the vast and growing number of procedures available nowadays, and even fewer assessing MIGS against topical medications. More comparative data, especially with gold standard therapies and common practice options could be extremely relevant for ophthalmologists and healthcare authorities, allowing to ascertain the best therapeutic option for the patients, and potentially reducing the medication burden and its associated costs. But considerably more evidence will be needed to achieve this level of certainty. Indeed, current evidence, while non-negligible, is still mostly limited to non-randomized studies and uncontrolled retrospective comparisons with few quality randomized controlled trials. This leads to significant variability in studies' results and a blurring of the outcomes, and further highlights the need for carefully designed randomized controlled trials.

Beyond the scope of MIGS, the potential of IOP telemetry for self-assessment of IOP control through implantable sensors is developing into a real option for clinicians and an empowering opportunity for patients. Indeed, providing patients with direct feedback enables them to take control and have a clearer representation of their care, in turn leading to a better control of the disease.^[68]^ Building on the reports that patients' adherence increases shortly before their medical appointments, continuous measurements may provide patients with an incentive for better adherence to treatment, and clinicians with a more accurate representation of out-of-clinic IOPs. It also might remove an element of self-deception where brief periods of adherence suffice to reassure both patients and treating clinicians.^[53]^ This paves the way for personalized glaucoma care, in which the actual effect of lifestyle and treatments can be self-monitored by the patients and therapeutic decisions can be made accordingly in real time before there is further optic nerve injury.

Patients are increasingly demanding an equal role in clinical decision-making. Many no longer accept the paternalistic model of medical care in which doctors decide and patients comply. However, there are potential issues with self-monitoring of IOP, such as increased anxiety levels induced by measured IOP fluctuations and peaks, leading to patients self-treating during IOP spikes and additional office visits. The availability of continual IOP monitoring will be a challenge to the current glaucoma management paradigm, which, however, has the important potential to improve patient health outcomes.

After several decades of relative stagnation through the last century, glaucoma has now entered what many view as a golden age for the specialty. Like every revolution, this one brings its fair share of uncertainty, clinical questioning, and uneasy periods of adaptation to ever-changing expectations. Yet, while it is impossible to guess what the landscape of glaucoma surgery will be like in 10 or 15 years, data suggest a bright outlook both for patients and clinicians.

##  Financial Support and Sponsorship

This review has been supported in part by the Swiss Glaucoma Research Foundation, Lausanne, Switzerland.

##  Conflicts of Interest

There are no conflicts of interest.
